# Association of Uric Acid–Lowering Therapy With Incident Chronic Kidney Disease

**DOI:** 10.1001/jamanetworkopen.2022.15878

**Published:** 2022-06-03

**Authors:** Waleed Hassan, Prabin Shrestha, Keiichi Sumida, Fridtjof Thomas, Patrick L. Sweeney, Praveen K. Potukuchi, Connie M. Rhee, Elani Streja, Kamyar Kalantar-Zadeh, Csaba P. Kovesdy

**Affiliations:** 1 Department of Medicine, North Mississippi Medical Center, Tupelo; 2 Division of Nephrology, University of Tennessee Health Science Center, Memphis; 3 Department of Preventive Medicine, University of Tennessee Health Science Center, Memphis; 4 John W. Demming Department of Medicine, Tulane University School of Medicine, New Orleans, Louisiana; 5 Harold Simmons Center for Chronic Disease Research and Epidemiology, Division of Nephrology and Hypertension, University of California–Irvine, Orange; 6 Long Beach VA Medical Center, Long Beach, California; 7 Nephrology Section, Memphis VA Medical Center, Memphis, Tennessee

## Abstract

**Importance:**

Uric acid is a waste metabolite produced from the breakdown of purines, and elevated serum uric acid levels are associated with higher risk of hypertension, cardiovascular disease, and mortality and progression of chronic kidney disease (CKD). Treatment of hyperuricemia in patients with preexisting CKD has not been shown to improve kidney outcomes, but the associations of uric acid–lowering therapies with the development of new-onset kidney disease in patients with estimated glomerular filtration rate (eGFR) within reference range and no albuminuria is unclear.

**Objective:**

To examine the association of initiating uric acid–lowering therapy with the incidence of CKD.

**Design, Setting, and Participants:**

This cohort study included patients with eGFR of 60 mL/min/1.73 m^2^ or greater and no albuminuria treated at US Department of Veterans Affairs health care facilities from 2004 to 2019. Clinical trial emulation methods, including propensity score weighting, were used to minimize confounding. Data were analyzed from 2020 to 2022.

**Exposure:**

Newly started uric acid–lowering therapy.

**Main Outcomes and Measures:**

The main outcomes were incidences of eGFR less than 60 mL/min/1.73 m^2^, new-onset albuminuria, and end-stage kidney disease.

**Results:**

A total of 269 651 patients were assessed (mean [SD] age, 57.4 [12.5] years; 252 171 [94%] men). Among these, 29 501 patients (10.9%) started uric acid–lowering therapy, and 240 150 patients (89.1%) did not. Baseline characteristics, including serum uric acid level, were similar among treated and untreated patients after propensity score weighting. In the overall cohort, uric acid–lowering therapy was associated with higher risk of both incident eGFR less than 60 mL/min/1.73 m^2^ (weighted subhazard ratio [SHR], 1.15 [95% CI, 1.10-1.20; *P* < .001) and incident albuminuria (SHR, 1.05 [95% CI, 1.01-1.09; *P* < .001) but was not associated with the risk of end-stage kidney disease (SHR, 0.96 [95% CI, 0.62-1.50]; *P* = .87). In subgroup analyses, the association of uric acid–lowering therapy with worse kidney outcomes was limited to patients with baseline serum uric acid levels of 8 mg/dL or less.

**Conclusions and Relevance:**

These findings suggest that in patients with kidney function within reference range, uric acid–lowering therapy was not associated with beneficial kidney outcomes and may be associated with potential harm in patients with less severely elevated serum uric acid levels.

## Key Points

**Question:**

What is the association of uric acid–lowering therapy with the development of new onset chronic kidney disease (CKD)?

**Findings:**

In this cohort study of 269 651 patients with estimated glomerular filtration rate of at least 60 mL/min/1.73 m^2^ and no albuminuria, there was no beneficial association between initiating uric acid–lowering therapy and the incidence of CKD. Uric acid lowering–therapy was associated with a significantly higher risk of new-onset CKD.

**Meaning:**

These findings do not support the initiation of uric acid–lowering therapy as a means to prevent the development of CKD.

## Teaser

This cohort examines the association of initiating uric acid–lowering therapy with the incidence of chronic kidney disease among patients receiving care at US Department of Veterans Affairs health care facilities.

## Introduction

Chronic kidney disease (CKD) affects more than 10% of the US general population, and it is estimated that approximately 850 million people worldwide are affected by kidney diseases.^1,2^ The high incidence and prevalence of CKD has prompted efforts to identify treatable risk factors of kidney disease. Uric acid is a purine metabolite that has numerous deleterious physiologic effects, including vascular smooth muscle cell proliferation, inhibition of nitric oxide production, endothelial dysfunction, and oxidative stress.^3,4,5,6,7^ These could translate to harmful organ effects, potentially explaining the association of elevated urate levels with all-cause mortality, ischemic stroke, myocardial infarction, and congestive heart failure in observational studies.^8,9,10,11,12^ Additionally, higher uric acid level is associated with hypertension,^13,14^ which could potentially explain a putative deleterious effect on the kidneys, in addition to direct effects on kidney structures.^15,16,17^ The association of elevated urate level with progression of preexisting CKD has been documented,^18,19,20,21,22,23^ but the casual link between kidney function and uric acid level is complex, as decreased kidney function itself can result in hyperuricemia. Higher urate level was also associated with a higher incidence of CKD in previously healthy individuals in a meta-analysis,^24^ but individual observational studies have shown disparate results.^25,26,27,28^

A causal effect of uric acid on CKD could be proven by demonstrating an impact of therapeutic urate lowering on kidney outcomes. Recent clinical trials of urate lowering in patients with existing CKD did not document a reduction in CKD progression,^29,30^ but the effects of urate lowering on the incidence of new-onset CKD are less clear. We examined the association of uric acid–lowering therapy with the incidence of various kidney outcomes in a large cohort of US veterans with no preexisting CKD. We hypothesized that lowering urate levels is independently associated with a lower incidence of new-onset kidney disease.

## Methods

This cohort study was approved by the institutional review boards of the Memphis and Long Beach VA Medical Centers, with exemption from informed consent because there was minimal risk for cohort participants. This report follows the Strengthening the Reporting of Observational Studies in Epidemiology (STROBE) reporting guideline for observational studies.

### Cohort Definition

**Figure 1.**
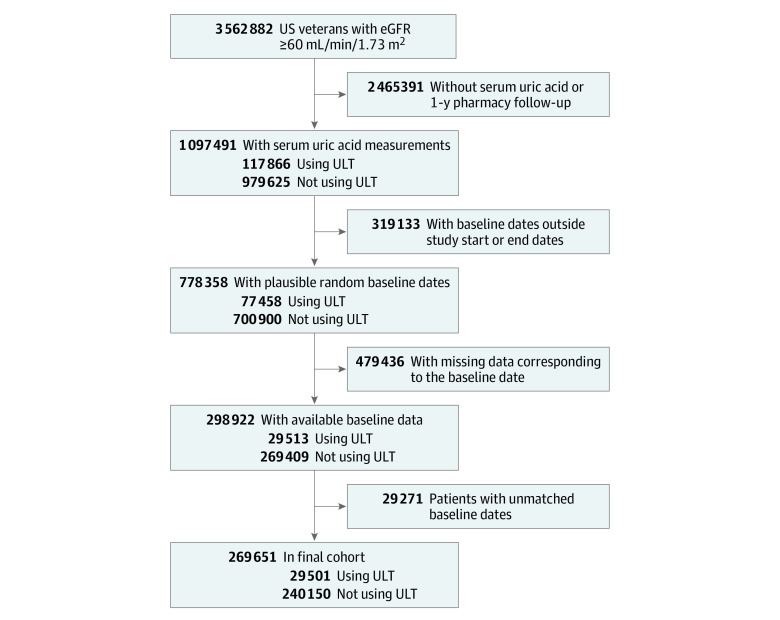
Flowchart of Cohort Creation eGFR, estimated glomerular filtration rate; ULT, uric acid–lowering therapy.

We analyzed data from the Therapeutic Interventions in Chronic Kidney Disease (TRI-CKD) study, a retrospective cohort study of 3 562 882 US veterans with estimated glomerular filtration rate (eGFR) of 60 mL/min/1.73 m^2^ or greater recorded from October 1, 2004, through September 30, 2006, and followed up until September 30, 2019. We identified 1 097 491 patients who had at least 1 serum uric acid level measured after the original cohort entry date and who had documented dispensations from a Department of Veterans Affairs (VA) pharmacy (Figure 1; eFigure 1 in the Supplement). Among these, we identified 117 866 patients (10.7%) newly initiated on any type of urate lowering–therapy after the first serum uric acid measurement and throughout the entire follow-up period and 979 625 patients (89.3%) who did not receive such treatment. We excluded 319 133 patients who developed a study end point prior to baseline (ie, the start date of urate lowering–therapy, or a randomly generated baseline date for untreated patients). Finally, we excluded 479 436 patients with missing data corresponding to the baseline date and 29 271 patients whose baseline date could not be matched to the same 180-day calendar period between treated and untreated patients. The final study sample included 269 651 patients with eGFR of 60 mL/min/1.73 m^2^ or greater and no albuminuria (29 501 treated patients [10.9%]; 240 150 untreated patients [89.1%]) in our primary analyses.

### Data Collection

We collected data on demographic and socioeconomic characteristics, comorbidities, medications, vital signs, and laboratory characteristics corresponding to each patient’s baseline date through the VA Corporate Data Warehouse (CDW). We used race and ethnicity categories as reported in the VA CDW, including African American, Hispanic, White, and other, which included those who self-identified as Asian, American Indian, Pacific Islander, and other without further specification. Race and ethnicity data were included because they have known associations with kidney outcomes and are thus considered confounders. We collected information about prescribed medications from the Decision Support System National Data Extracts’ outpatient and inpatient pharmacy files, including the date of dispensation, the dose, and the number of pills, as well as from Medicare Part D files for those eligible for such coverage.^31^ We identified medications obtained outside VA pharmacies from non-VA medication files in CDW. We defined baseline medication use as the presence of at least 1 outpatient prescription for at least 30 days during the 365 days preceding the baseline date. We extracted information about comorbidities from the VA Inpatient and Outpatient Medical SAS Data sets, using *International Classification of Diseases, Ninth Revision* (*ICD-9*) and *International Statistical Classification of Diseases and Related Health Problems, Tenth Revision* (*ICD-10*) diagnostic and procedure codes and *Current Procedural Terminology* (CPT) codes, as well as from Centers for Medicare & Medicaid Services (CMS) data files, and defined prevalent comorbid conditions based on the presence of at least 1 inpatient code or at least 2 outpatient codes recorded prior to the baseline date. We calculated the Charlson Comorbidity Index score using the Deyo modification for administrative data sets.^32^ We collected information about relevant laboratory characteristics from the VA LabChem files^33^ and we calculated eGFR using the Chronic Kidney Disease Epidemiology Collaboration (CKD-EPI) equation.^34^ We collected information about proteinuria, including urine protein to creatinine ratio, urine albumin to creatinine ratio (UACR), and urine dipstick protein, from the DSS National Data Extracts Laboratory Results file and the VA LabChem file in the CDW. Since UACR is the preferred method for defining and staging CKD, we converted urine protein to creatinine ratio and urine dipstick protein to UACR values using the conversion equations by Sumida et al^35^ and categorized all available UACR values as less than 30 mg/g (absence of albuminuria) or 30 mg/g or greater (presence of albuminuria).

### Exposure, Outcomes, and Study Design

We used an incident new user design to define treatment exposure. We defined exposure as the de novo initiation of any chronic oral uric acid–lowering therapy (ie, allopurinol, febuxostat, or probenecid) following the first measurement of serum uric acid level and throughout the entire follow-up period. Chronic new users were defined as patients receiving a first-time outpatient prescription of uric acid–lowering therapy covering at least 30 days, preceded by the lack of any prescriptions of urate lowering agents during the previous 365 days while having a record of VA pharmacy enrollment during the same period. We defined controls as patients who did not receive uric acid–lowering therapy but had at least 1 serum uric acid level measured.

Our coprimary outcomes were the incidences of new-onset eGFR less than 60 mL/min/1.73 m^2^ and of new-onset albuminuria. Both of these had to be measured twice with at least 90 days between the 2 measurements, with the outcome date corresponding to the date of the first measurement. For the incidence of eGFR less than 60 mL/min/1.73 m^2^, we also specified that the values had to be at least 25% lower than the baseline eGFR.^36^ We also examined end-stage kidney disease (ESKD), defined as the initiation of kidney replacement therapy (ie, dialysis or preemptive kidney transplant) and identified from the US Renal Data System.^37^ We considered all-cause death as a competing risk, identified from the VA Vital Status Files.^38^ Patients were followed up until the occurrence of any of these outcomes, last recorded VA encounter date, or end of follow-up (September 30, 2019, or June 30, 2018 [for the ESKD outcome only]).

We examined the association of urate-lowering therapies (vs no therapies) with the outcomes of interest using a target trial emulation with an intention-to-treat–like design. We used the intention-to-treat principle from randomized trials (the treatment exposure at cohort entry is carried forward irrespective of future treatment status) to model a trial in which patients initiating de novo urate-lowering therapy are compared with untreated comparators who are included (as a simulation of trial recruitment) within the same 180-day calendar period. We described actual exposure to uric acid–lowering therapy in the treated group by calculating the proportion of days covered (PDC; the proportion of days during which a treated individual had access to the drug during the follow-up period). Treated patients started follow-up on the date of receiving the first de novo prescription, while untreated patients entered follow-up on an assigned date randomly chosen based on the start of follow-up dates in the treatment exposure group, occurring after the measurement of a first serum uric acid level but before the occurrence of a potential outcome or censoring date, with the exclusion of patients whose randomly assigned entry dates fell outside of these dates.

### Statistical Analysis

Data are presented as number with percentage for categorical variables and mean with SD or median with IQR for continuous variables. Comparisons between characteristics in patients with and without treatment with uric acid–lowering therapies were performed using standardized differences, with differences higher than 10% considered to be significant. Hypothesis tests were 2-sided. We calculated cumulative incidence rates for all outcomes (per 1000 patient-years [PY]) overall and stratified by treatment status, incorporating the competing risk of death. We examined the association of uric acid–lowering treatment (vs no treatment) with the events of interest in competing risk regression models using the Fine and Gray method,^39^ with mortality as the competing event. We accounted for differences between baseline characteristics using propensity scores (PSs) calculated from logistic regression models, using as variables the 180-day baseline time period, patient baseline age, sex, race, ethnicity, marital status, insurance type, baseline use of medications (ie, renin-angiotensin system inhibitors, potassium sparing diuretics and mineralocorticoid receptor antagonists, thiazide diuretics, loop diuretics, other blood pressure–lowering agents, sodium glucose cotransporter 2 inhibitors, proton pump inhibitors, nonsteroidal anti-inflammatory agents, and opioid analgesics), comorbidities (ie, diabetes, myocardial infarction, peripheral vascular and cerebrovascular disease, congestive heart failure, and the Charlson Comorbidity Index), body mass index, systolic and diastolic blood pressure, baseline eGFR, and serum uric acid level. Owing to the presence of extreme PSs (eFigure 2 in the Supplement), we used the PS overlap weighting method^40,41^ as our primary approach. In a sensitivity analysis, we also applied PS matching using 1-to-1 nearest-neighbor matching and examined associations in unadjusted models and after adjustment for all variables used to calculate the propensity scores. We examined heterogeneity of treatment outcomes from important patient characteristics by examining associations in subgroup analyses for the outcomes of incident eGFR less than 60 mL/min/1.73 m^2^ and incident albuminuria. There were too few ESKD events to allow meaningful subgroup analyses.

Analyses were conducted using Stata MP statistical software version 17.1 (StataCorp) and SAS statistical software version 9.4 (SAS Institute). Analyses were conducted in 2020 to 2022.

## Results

### Baseline Characteristics

We identified 269 651 patients in our primary analytical cohort. The mean (SD) age was 57.4 (12.5) years, and 252 171 patients (94%) were men. The sample included 49 932 African American patients (19%), 25 274 Hispanic patients (9%), and 203 643 White patients (77%). A total of 66 276 patients (25%) had diabetes (Table 1). Of 29 501 patients treated with urate lowering therapy, 29 422 patients (99.7%) received allopurinol. The mean (SD) PDC for urate-lowering therapy during the follow-up period in treated patients was 0.79 (0.38). Patients initiating uric acid–lowering therapy were more likely to be men and African American, to be treated with various medications, to have higher blood pressure and body mass index, and to have lower eGFR and higher serum uric acid concentration (Table 1). The baseline characteristics of patients receiving and not receiving uric acid–lowering therapy were similar after PS weighting (standardized differences <0.1) (Table 1) and after PS matching (eTable 1 in the Supplement). The mean (SD) serum uric acid concentration during follow-up was 6.4 (1.6) mg/dL in the treated group vs 7.0 (1.4) mg/dL in the untreated group (to convert to millimoles per liter, multiply by 0.0595).

**Table 1.** Baseline Characteristics of the Overall Cohort and of Patients Receiving and Not Receiving Uric Acid–Lowering Therapy

Characteristic	Patients, No. (%)			Standardized difference	
	All (N = 269 651)	Uric acid–lowering therapy (n = 29 501)	No uric acid–lowering therapy (n = 240 150)	Unweighted	PS overlap weighted
Age, mean (SD), y	57.4 (12.5)	56.4 (10.6)	57.6 (12.8)	–0.0984	0.0178
Sex					
Men	252 171 (94)	28 928 (98)	223 243 (93)	0.2480	0.0059
Women	17 480 (6)	573 (2)	16 907 (7)		
Race					
African American	49 932 (19)	7270 (25)	42 662 (18)	0.1720	<0.0001
White	203 643 (77)	20 643 (71)	183 000 (78)		
Other^a^	9940 (4)	1104 (4)	8836 (4)		
Hispanic ethnicity	25 274 (9)	1661 (6)	23 613 (10)	–0.1590	0.0010
Marital status					
Single	27 972 (10)	2632 (8.9)	25 340 (11)	0.1669	<0.0001
Married	144 123 (53)	16 085 (55)	128 038 (53)		
Divorced	75 643 (28)	8747 (30)	66 896 (28)		
Widowed	17 470 (7)	1696 (6)	15 774 (7)		
Unknown	4443 (2)	341 (1)	4102 (2)		
Service connected	158 457 (59)	18 336 (62)	140 121 (58)	0.0778	–0.0011
Insurance type					
None	122 908 (46)	13 142 (45)	109 766 (46)	0.0277	<0.0001
Medicare	93 946 (35)	10 094 (34)	83 852 (35)		
Other	52 664 (20)	6261 (21)	46 403 (19)		
Medications					
RAAS inhibitors	153 279 (57)	21 072 (71)	132 207 (55)	0.3447	0.0096
Thiazide diuretics	110 114 (41)	16 444 (56)	93 670 (39)	0.3400	0.0025
Loop diuretics	52 039 (19)	8150 (28)	43 889 (18)	0.2237	0.0061
Potassium sparing diuretics	24 018 (9)	3879 (13)	20 139 (8)	0.1541	0.0053
Other antihypertensives	155 977 (58)	20 164 (68)	135 813 (57)	0.2454	0.0097
NSAIDs	190 584 (71)	24 508 (83)	166 076 (69)	0.3309	0.0064
Opioid	187 515 (70)	23 467 (80)	164 048 (68)	0.2580	0.0159
SGLT2 inhibitors	3964 (2)	469 (2)	3495 (2)	0.0110	0.0008
Proton pump inhibitors	144 972 (54)	17 724 (60)	127 248 (53)	0.1434	0.0034
Comorbidities					
Myocardial infarction	14 275 (5)	1754 (6)	12 521 (5)	0.0319	0.0003
Congestive heart failure	15 275 (5)	2339 (8)	12 936 (5)	0.1021	0.0038
Cerebrovascular disease	19 260 (7)	1919 (7)	17 341 (7)	–0.0283	0.0003
Peripheral vascular disease	18 509 (7)	1990 (7)	16 519 (79)	–0.0053	0.0011
Diabetes	66 276 (25)	8066 (27)	58 210 (24)	0.0710	0.0054
Charlson comorbidity index, median (IQR)	1 (0-2)	1 (0-2)	1 (0-2)	0.0318	0.0094
BMI, mean (SD)	29.9 (5.9)	32.2 (6.2)	29.6 (5.8)	0.4411	–0.0018
Blood pressure, mean (SD), mm Hg					
Systolic	129.8 (15.7)	131.4 (15.7)	129.6 (15.7)	0.1183	–0.0034
Diastolic	76.2 (10.6)	77.9 (10.9)	76 (10.6)	0.1763	–0.003
eGFR, mean (SD), mL/min/1.73 m^2^	85.6 (14.9)	82.3 (14.2)	86 (14.9)	–0.2578	–0.0005
Serum uric acid, mean (SD), mg/dL	6.0 (1.6)	8.0 (1.7)	5.7 (1.4)	1.5142	0.0057

Abbreviations: BMI, body mass index (calculated as weight in kilograms divided by height in meters squared); eGFR, estimated glomerular filtration rate; NSAID, nonsteroidal anti-inflammatory drug; PS, propensity score; RAAS, renin-angiotensin aldosterone system; SGLT2, sodium glucose cotransporter 2.

SI conversion factor: To convert serum uric acid to millimoles per liter, multiply by 0.0595.

^a^ Includes individuals who identified as Asian, American Indian, or Pacific Islander and those who identified as other race or ethnicity without providing further information.

### Association of Urate Lowering Therapy With Kidney Outcomes

**Figure 2.**
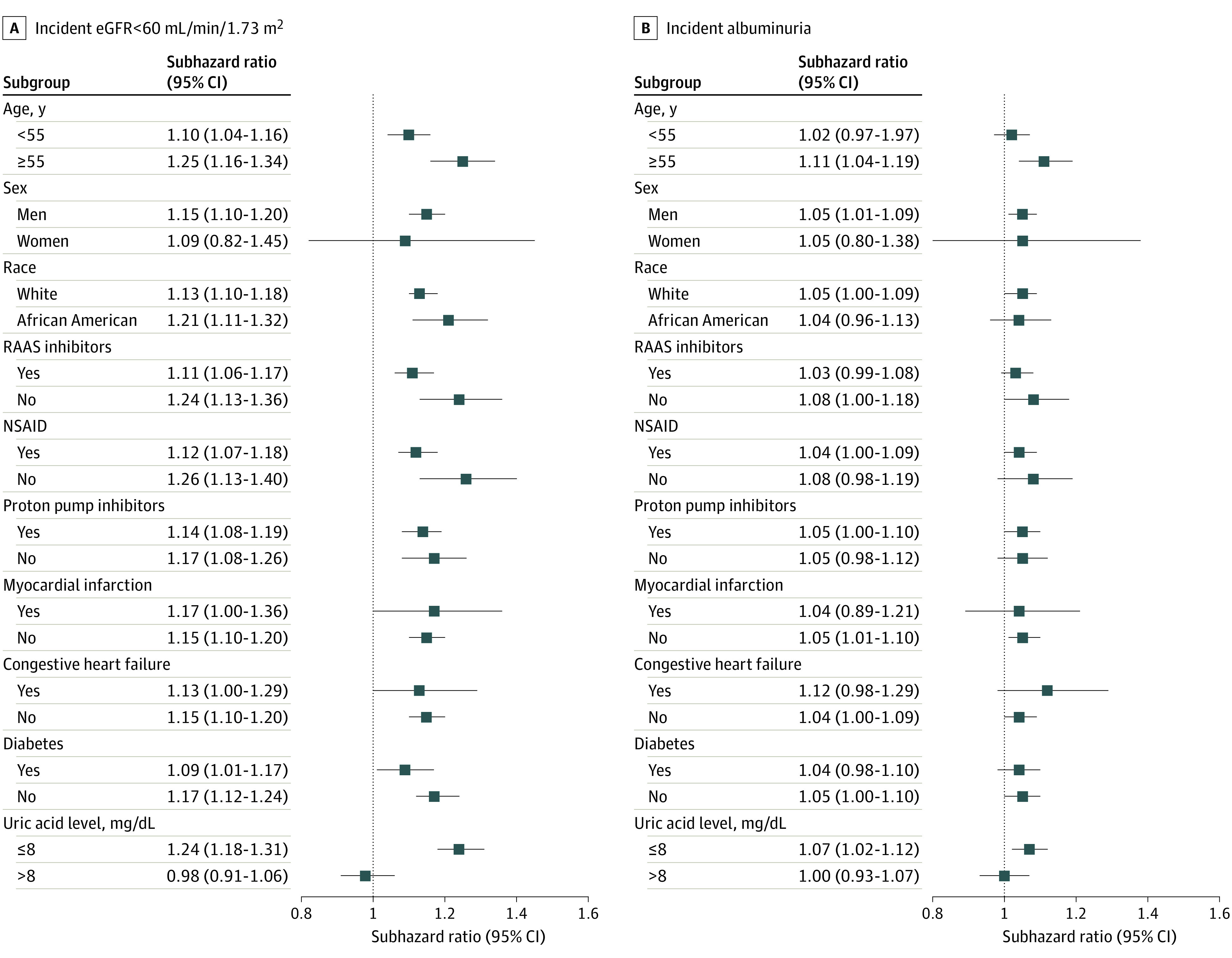
Hazard of Incident Estimated Glomerular Filtration Rate (eGFR) Less Than 60 mL/min/1.73 m^2^ and Albuminuria Associated With Uric Acid–Lowering Therapy in Propensity Score–Weighted Analyses To convert uric acid to millimoles per liter, multiply by 0.0595. RAAS indicates renin angiotensin aldosterone system; NSAID, nonsteroidal anti-inflammatory drugs.

A total of 58 481 patients (21.7%) experienced an incident eGFR less than 60 mL/min/1.73 m^2^ (event rate, 29.8 [95% CI, 29.56-30.03] per 1000 PY), 68 759 patients (25.5%) experienced incident albuminuria (event rate, 36.31 [95% CI, 36.04-36.57] per 1000 PY), and 531 patients (0.2%) experienced incident ESKD (event rate, 0.26 [95% CI, 0.24-0.29] per 1000 PY). Event rates and unadjusted subhazard ratios (SHRs) for all 3 outcomes were higher in patients initiating urate lowering therapy (Table 2; eTable 2 and eFigure 3 in the Supplement). In the PS-weighted cohorts, initiation of uric acid–lowering therapy was associated with significantly higher risk of incident eGFR less than 60 mL/min/1.73 m^2^ (SHR, 1.15 [95% CI, 1.10-1.20]; *P* < .001) and incident albuminuria (SHR, 1.05 [95% CI, 1.01-1.09]; *P* < .001) but was not associated with ESKD (Table 2). Associations of uric acid–lowering treatment with incident eGFR less than 60 mL/min/1.73 m^2^ and incident albuminuria were similar when examined in subgroups of age, sex, race, use or nonuse of various medication classes, and presence or absence of diabetes, congestive heart failure, or myocardial infarction (Figure 2). The association of urate-lowering therapy with kidney outcomes was different in subgroups divided by baseline serum uric acid concentration, with a higher risk of both outcomes observed in patients with uric acid levels of 8 mg/dL or less and no significant association observed in patients with uric acid levels greater than 8 mg/dL (Figure 2). Results remained similar when examined in a cohort of 50 206 PS-matched patients, with 25 118 patients in both treatment groups (Table 2; eFigure 4 in the Supplement).

**Table 2.** Outcomes Associated With Uric Acid–Lowering Therapy Compared With No Uric Acid–Lowering Therapy

Event	Primary cohort				PS-matched cohort			
	Crude model		PS overlap weighted model		Crude model		Multivariable adjusted model	
	SHR (95% CI)	P value	SHR (95% CI)	P value	SHR (95% CI)	P value	SHR (95% CI)	P value
Incident eGFR <60 mL/min/1.73 m^2^	1.34 (1.30-1.37)	<.001	1.15 (1.10-1.20)	<.001	1.10 (1.07-1.14)	<.001	1.10 (1.06-1.14)	<.001
Incident albuminuria	1.29 (1.26-1.32)	<.001	1.05 (1.01-1.09)	<.001	1.04 (1.00-1.08)	.01	1.03 (1.00-1.07)	.05
ESKD	1.74 (1.37-2.21)	<.001	0.96 (0.62-1.50)	.87	0.86 (0.62-1.21)	.39	0.79 (0.56-1.10)	.16

Abbreviations: eGFR, estimated glomerular filtration rate; ESKD, end-stage kidney disease; PS, propensity score; SHR, subhazard ratio.

## Discussion

In this large national cohort study of US veterans, uric acid–lowering therapy was not associated with improved kidney outcomes, including the incidence of eGFR less than 60 mL/min/1.73 m^2^, albuminuria, or ESKD. Contrary to our hypothesis, we found that uric acid–lowering therapy (which was predominantly achieved with the administration of allopurinol in our cohort) was associated with a higher incidence of new-onset CKD and showed no association with incident ESKD. In subgroup analysis, the association of uric acid–lowering therapy with unfavorable kidney outcomes was limited to patients with baseline serum uric acid levels of 8 mg/dL or less, while the same associations were not significant in patients with serum uric acid levels greater than 8 mg/dL. These results do not support a direct benefit of urate lowering on the development of new-onset CKD, and support the results of recent large randomized clinical trials^29,30^ that found no benefit of allopurinol in delaying progression of established CKD. Our study is notable for its large size, national representativeness, and availability of comprehensive information on a broad array of clinical data.

Elevated serum uric acid can be lowered using readily available therapies; hence, the impact of uric acid level on various clinical outcomes can be studied in clinical trials using such treatments. Smaller trials have suggested benefits from lowering serum uric acid levels in patients with preexisting kidney disease,^42,43,44,45^ but 2 large randomized clinical trials examining reducing uric acid in patients with type 1 diabetes and in patients without diabetes found that treatment of hyperuricemia did not result in improved progression of preexisting kidney disease.^29,30^ The impact of urate-lowering therapies on the incidence of new-onset CKD has been less well studied in clinical trials. While some have suggested that uric acid lowering is beneficial in preventing the development of CKD,^46,47^ the quality of these trials has been low; hence, their results are not conclusive.

Our findings of higher risk of incident CKD and albuminuria in patients with less severe elevations of serum uric concentration treated with urate lowering therapy may appear surprising, as we hypothesized that the lowering of uric acid levels would be beneficial owing to the detrimental effects of uric acid on various metabolic and cardiovascular processes. While it is possible that the higher risk associated with urate-lowering therapy was due to residual confounding, we cannot exclude the possibility that the administration of allopurinol (the uric acid–lowering agent used in most of our cohort) could also be deleterious. Allopurinol has a known potential to cause acute allergic reactions and could cause acute kidney injury by inducing acute interstitial nephritis.^48^ We used a robust definition of incident CKD, which is less likely to be affected by transient elevations of serum creatinine, but acute kidney injury events could potentially contribute to the development of CKD, thus explaining our findings.

### Limitations

Our study has several limitations that should be considered when interpreting its results. We examined mostly US veteran men; hence, it is unclear if our results apply to women or nonveterans in general. Our study is observational and retrospective and therefore prone to confounding. While we accounted for major known confounders of the development of kidney disease, residual confounding remains possible, such as the doses of various potentially nephrotoxic medications or the severity of cardiovascular disease or other comorbidities. We used data collected from multiple hospitals during an extended period. While all of the hospitals currently use the isotope-dilution mass spectrometry traceable method to measure serum creatinine, we cannot ascertain when all these hospitals made the transition to this method, which may have resulted in misclassification of presence or absence of CKD. In our attempt to examine treated and untreated patients with comparable characteristics, we excluded a large number of patients, thus limiting the external validity of our findings to patients with characteristics similar to those included in our analyses.

## Conclusions

This cohort study found that uric acid–lowering therapy was not associated with beneficial kidney outcomes, including the incidence of eGFR less than 60 mL/min/1.73 m^2^, albuminuria, or ESKD. Uric acid–lowering therapy was associated with a higher risk of new-onset CKD, including both the development of eGFR less than 60 mL/min/1.73m^2^ and new-onset albuminuria, in patients with baseline serum uric acid levels of 8 mg/dL or lower. The causal effect of uric acid–lowering therapies, and especially allopurinol, in patients with no preexisting CKD needs to be examined in properly powered randomized clinical trials. Short of such trials, the preponderance of existing evidence does not support the administration of uric acid–lowering therapies as a means to prevent the development of CKD.

## Notes

Supplement. **eTable 1.** Baseline Characteristics of Propensity Score–Matched Cohort **eTable 2.** Event Numbers and Event Rates in the Primary and Propensity Score Matched Cohorts **eFigure 1.** Schematic of Study Cohort **eFigure 2.** Propensity Score Distribution **eFigure 3.** Cumulative Incidence Function for Incident CKD in Patients Treated and Not Treated With Uric Acid–Lowering Therapy in the Propensity Score–Matched Cohort **eFigure 4.** Hazard of Incident eGFR Less Than 60 mL/min/1.73 m^2^ and Incident Albuminuria Associated With Uric Acid Lowering Therapy in the Propensity Score–Matched Cohort
