# Supplementary material for: Association of Uric Acid–Lowering Therapy With Incident Chronic Kidney Disease
Source: JAMA Netw Open. 2022 Jun 3;5(6):e2215878. doi: 10.1001/jamanetworkopen.2022.15878 (PMC9166229; doi:10.1001/jamanetworkopen.2022.15878)
Supplement: Supplement. — eTable 1. Baseline Characteristics of Propensity Score–Matched Cohort eTable 2. Event Numbers and Event Rates in the Primary and Propensity Score Matched Cohorts eFigure 1. Schematic of Study Cohort eFigure 2. Propensity Score Distribution eFigure 3. Cumulative Incidence Function for Incident CKD in Patients Treated and Not Treated With Uric Acid–Lowering Therapy in the Propensity Score–Matched Cohort eFigure 4. Hazard of Incident eGFR Less Than 60 mL/min/1.73 m2 and Incident Albuminuria Associated With Uric Acid Lowering Therapy in the Propensity Score–Matched Cohort [file jamanetwopen-e2215878-s001.pdf]

Supplemental Online Content

Hassan W, Shrestha P, Sumida K, et al. Association of uric acid–lowering therapy with incident chronic kidney disease. *JAMA Netw Open*. 2022;5(6):e2215878. doi:10.1001/jamanetworkopen.2022.15878

eTable 1. Baseline Characteristics of Propensity Score–Matched Cohort

eTable 2. Event Numbers and Event Rates in the Primary and Propensity Score Matched Cohorts

eFigure 1. Schematic of Study Cohort

eFigure 2. Propensity Score Distribution

eFigure 3. Cumulative Incidence Function for Incident CKD in Patients Treated and Not Treated With Uric Acid–Lowering Therapy in the Propensity Score–Matched Cohort

eFigure 4. Hazard of Incident eGFR Less Than 60 mL/min/1.73 m^2 and Incident Albuminuria Associated With Uric Acid Lowering Therapy in the Propensity Score–Matched Cohort

This supplemental material has been provided by the authors to give readers additional information about their work.

© 2022 Hassan W et al. *JAMA Network Open.*

eTable 1. Baseline Characteristics of Propensity Score–Matched Cohort

	All	Uric acid lowering therapy	No uric acid lowering therapy	Standardized difference
	(*n* = 50,236)	(*n* = 25,118)	(*n* = 25,118)	
Age (years)	56.9±11.1	56.8±10.5	57.9±11.7	0.00573
Gender (male)	49,081 (98)	24,594 (98)	24,487 (97)	0.02843
Race				-0.00059
White	36,482 (73)	18,196 (72)	18,286 (73)	
African-American	11,770 (23)	5,971 (24)	5,799 (23)	
Other	1,984 (4)	951 (3.8)	1,033 (4)	
Ethnicity (Hispanic)	2,920 (5.8)	1,442 (5.7)	1,478 (5.9)	-0.00613
Marital status				-0.00266
Single	4,597 (9.2)	2,147 (8.6)	2,450 (9.8)	
Married	27,393 (55)	13,879 (55)	13,514 (54)	
Divorced	14,602 (29)	7,333 (29)	7,269 (29)	
Widowed	3,017 (6)	1,477 (5.9)	1,540 (6.1)	
Unknown	627 (1.3)	282 (1.1)	345 (1.4)	
Service connected	30,956 (62)	15,488 (62)	15,468 (62)	-0.00164
Insurance type				0.01056
None	22,203 (44)	11,059 (44)	11,144 (44)	
Medicare	17,328 (34)	8,852 (35)	8,476 (34)	
Other	10,705 (21)	5,207 (21)	5,498 (22)	
RAAS inhibitors	35,000 (70)	17,570 (70)	17,430 (69)	-0.01213
Thiazide diuretics	27,037 (54)	13,598 (54)	13,439 (54)	-0.01270
Loop diuretics	13,136 (26)	6,523 (26)	6,613 (26)	0.00815
Potassium sparing diuretics	6,124 (12)	3,066 (12)	3,058 (12)	-0.00097
Other antihypertensives	33,761 (67)	16,908 (67)	16,853 (67)	-0.00466
NSAIDs	40,868 (81)	20,546 (82)	20,322 (81)	-0.02290
Opioid	39,304 (78)	19,691 (78)	19,613 (78)	-0.00753
SGLT2 inhibitors	790 (1.6)	402 (1.6)	388 (1.5)	-0.00448
Proton pump inhibitors	29,793 (59)	14,926 (59)	14,867 (59)	-0.00478
Myocardial infarction	2,973 (5.9)	1,487 (5.9)	1,486 (5.9)	-0.00017
Congestive heart failure	3,731 (7.4)	1,831 (7.3)	1,900 (7.6)	0.01048

© 2022 Hassan W et al. *JAMA Network Open.*

Cerebrovascular disease	3,427 (6.8)	1,677 (6.7)	1,750 (7)	0.01153
Peripheral vascular disease	3,401 (6.8)	1,699 (6.8)	1,702 (6.8)	0.00048
Diabetes mellitus	13,713 (27)	6,800 (27)	6,913 (28)	0.01010
Charlson comorbidity index*	1 (2)	1 (2)	1 (2)	0.04146
Body mass index (kg/m^2)	31.9±6.2	31.9±6	31.9±6.4	-0.00676
Systolic blood pressure (mmHg)	131.3±15.7	131.4±15.7	131.1±15.7	-0.01511
Diastolic blood pressure (mmHg)	77.6±10.8	77.7±10.8	77.6±10.8	-0.01192
Estimated GFR ($ml/min/1.73m^2$)	82.7±14.3	82.5±14.2	82.8±14.6	0.01744
Serum uric acid (mg/dl)	7.6±1.5	7.7±1.5	7.6±1.4	-0.08536

Results presented as number (percent) or means ± standard deviation.
RAAS, renin-angiotensin aldosterone system; NSAID, non-steroidal anti-inflammatory drug; SGLT2, sodium glucose co-transporter 2; GFR, glomerular filtration rate

© 2022 Hassan W et al. *JAMA Network Open.*

eTable 2. Event Numbers and Event Rates in the Primary and Propensity Score Matched Cohorts

Event	Primary cohort (*n* = 269,651)				PS matched cohort (*n* = 50,236)			
	Event numbers		Event rate (95%CI)		Event numbers		Event rate (95%CI)	
	ULT=0	ULT=1	ULT=0	ULT=1	ULT=0	ULT=1	ULT=0	ULT=1
Incident eGFR<60	50,930	7,551	29.4 (29.15, 29.66)	39.73 (38.83, 40.62)	6,344	6,453	34.7 (33.85, 35.56)	38.92 (37.99, 39.89)
Incident albuminuria	60,116	8,643	35.84 (35.55, 36.12)	46.56 (45.58, 47.54)	7,594	7,295	43.12 (42.16, 44.1)	45.01 (43.99, 46.06)
ESKD	452	79	0.26 (0.23, 0.28)	0.4 (0.31, 0.49)	85	60	0.45 (0.37, 0.56)	0.36 (0.28, 0.46)

Data presented as numbers and events /1000 patient-years (95% confidence intervals)

ULT: uric acid lowering therapy; PS, propensity score

© 2022 Hassan W et al. *JAMA Network Open.*

eFigure 1. Schematic of Study Cohort

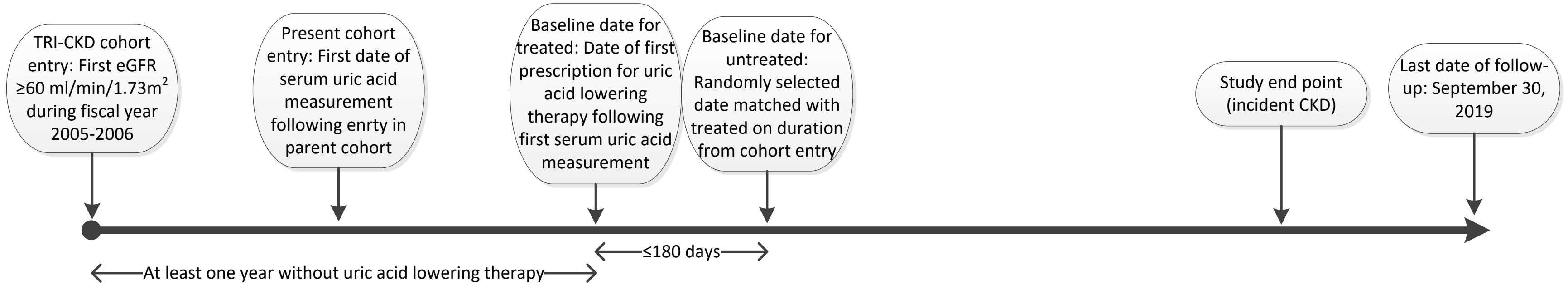

© 2022 Hassan W et al. *JAMA Network Open.*

eFigure 2. Propensity Score Distribution

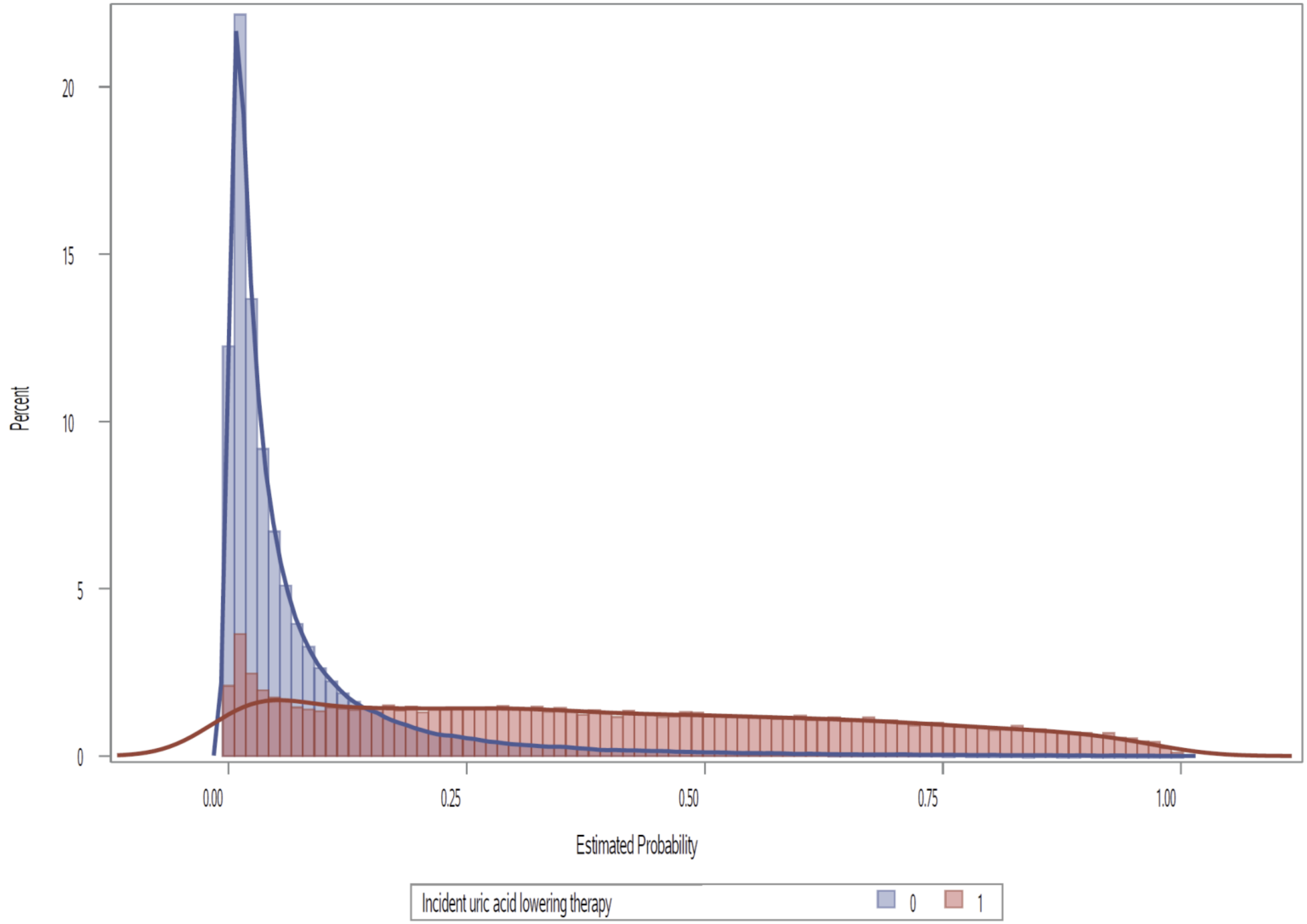

© 2022 Hassan W et al. *JAMA Network Open.*

eFigure 3. Cumulative Incidence Function for Incident CKD in Patients Treated and Not Treated With Uric Acid–Lowering Therapy in the Propensity Score–Matched Cohort

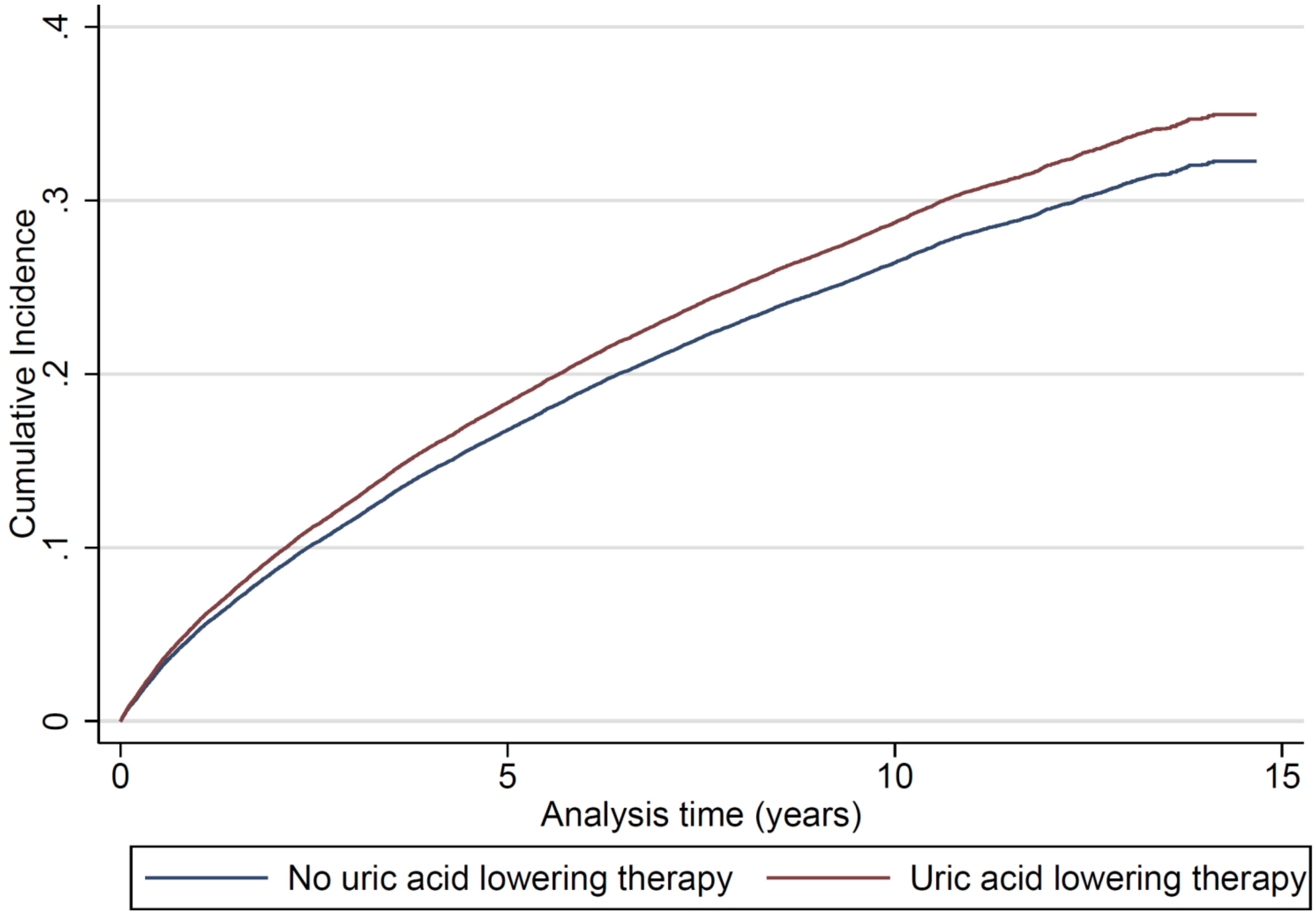

© 2022 Hassan W et al. *JAMA Network Open.*

eFigure 4. Hazard of Incident eGFR Less Than 60 mL/min/1.73 m2 and Incident Albuminuria Associated With Uric Acid Lowering Therapy in the Propensity Score–Matched Cohort

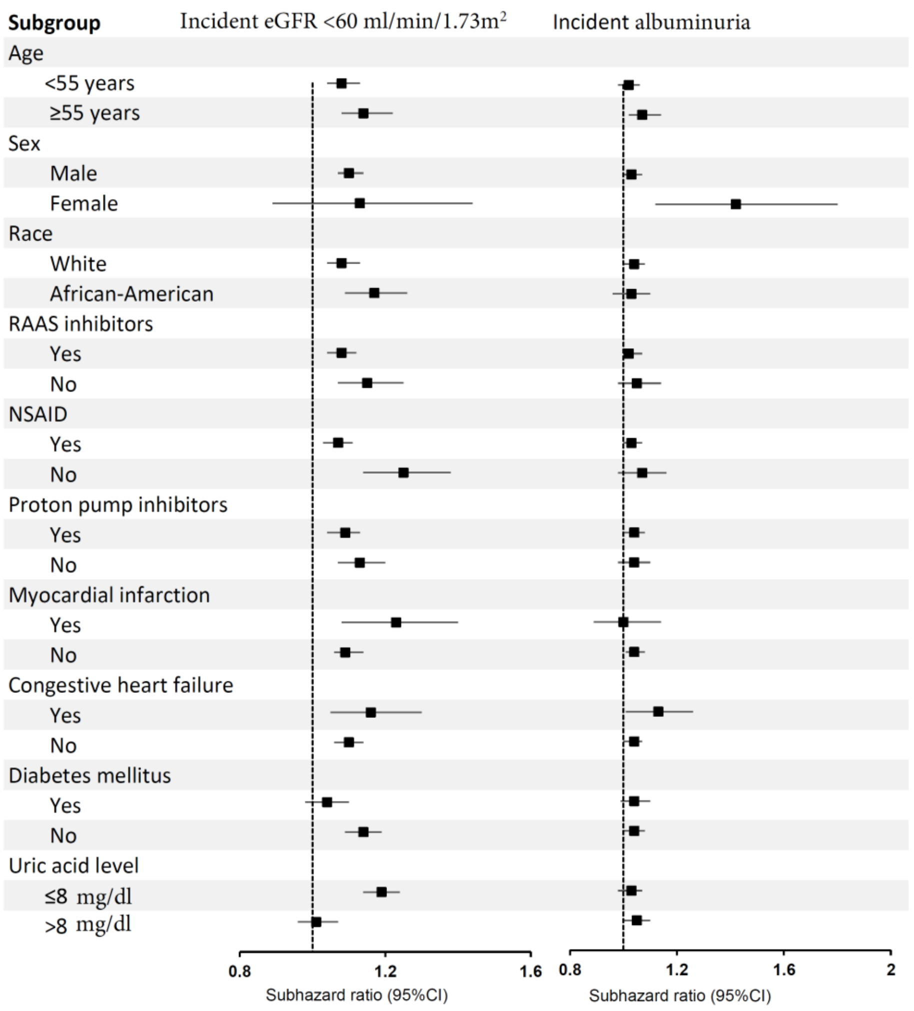

© 2022 Hassan W et al. *JAMA Network Open.*
